# *De Novo* Assembly and Characterization of Pericarp Transcriptome and Identification of Candidate Genes Mediating Fruit Cracking in *Litchi chinensis* Sonn.

**DOI:** 10.3390/ijms151017667

**Published:** 2014-09-30

**Authors:** Wei-Cai Li, Jian-Yang Wu, Hong-Na Zhang, Sheng-You Shi, Li-Qin Liu, Bo Shu, Qing-Zhi Liang, Jiang-Hui Xie, Yong-Zan Wei

**Affiliations:** 1 Key Laboratory of Tropical Fruit Biology (Ministry of Agriculture), South Subtropical Crops Research Institute, Chinese Academy of Tropical Agricultural Sciences, Zhanjiang 524091, China; E-Mails: lwc-619@163.com (W.-C.L.); meilideweihai@126.com (H.-N.Z.); ssy7299@163.com (S.-Y.S.); lolitallq@163.com (L.-Q.L.); bshbest@163.com (B.S.); qingzhi2002@163.com (Q.-Z.L.); xiejianghui@21cn.com (J.-H.X.); 2 Basic Education College of Zhanjiang Normal University, Zhanjiang 524037, China; E-Mail: wjiany065@163.com

**Keywords:** *Litchi chinensis* Sonn., transcriptome, pericarp, RNA-Seq, cracking

## Abstract

Fruit cracking has long been a topic of great concern for growers and researchers of litchi (*Litchi chinensis* Sonn.). To understand the molecular mechanisms underlying fruit cracking, high-throughput RNA sequencing (RNA-Seq) was first used for *de novo* assembly and characterization of the transcriptome of cracking pericarp of litchi. Comparative transcriptomic analyses were performed on non-cracking and cracking fruits. A total of approximately 26 million and 29 million high quality reads were obtained from the two groups of samples, and were assembled into 46,641 unigenes with an average length of 993 bp. These unigenes can be useful resources for future molecular studies of the pericarp in litchi. Furthermore, four genes (*LcAQP*, 1; *LcPIP*, 1; *LcNIP*, 1; *LcSIP*, 1) involved in water transport, five genes (*LcKS*, 2; *LcGA2ox*, 2; *LcGID1*, 1) involved in GA metabolism, 21 genes (*LcCYP707A*, 2; *LcGT*, 9; *Lcβ*-*Glu*, 6; *LcPP2C*, 2; *LcABI1*, 1; *LcABI5*, 1) involved in ABA metabolism, 13 genes (*LcTPC*, 1; *Ca^2+^/H^+^ exchanger*, 3; *Ca^2+^-ATPase*, 4; *LcCDPK*, 2; *LcCBL*, 3) involved in Ca transport and 24 genes (*LcPG*, 5; *LcEG*, 1; *LcPE*, 3; *LcEXP*, 5; *Lcβ-Gal*, 9; *LcXET*, 1) involved in cell wall metabolism were identified as genes that are differentially expressed in cracked fruits compared to non-cracked fruits. Our results open new doors to further understand the molecular mechanisms behind fruit cracking in litchi and other fruits, especially Sapindaceae plants.

## 1. Introduction

Litchi has been cultivated for more than 2300 years and is one of the most important tropical and subtropical plants of the Sapindaceae family. Litchi is widely grown in southern China and other Southeast Asian areas. Litchi production significantly contributes to the livelihood of millions of people and the economy of Southeast Asia. The production of litchi was 1.78 million tons from 553,000 ha in 2010 in China, where it is a major source of employment for the local people. The desirable characteristics of litchi fruit, such as its richness of nutrition, minerals, vitamins, phenolic compounds, and antioxidants, as well as its bright color, exotic aroma and good medicinal value, make it very attractive and popular in international markets. However, fruit cracking during growth and development of litchi is a serious problem that causes significant loss of yield and commercial value [1].

Cracking has been described as “the physical failure of the fruit skin” and occurs in the form of fractures in the cuticle or skin that typically do not penetrate the flesh. Cracking of fruits has been observed in many species, including pomegranate (*Punica granatum*) [2], wax apple (*Syzygium samarangense*) [3], fresh fig (*Ficus carica* L.) [4], cherry tomato (*Solanum lycopersicum* var. *cerasiforme*) [5], nectarine fruit (*Prunus persica* L.) [6], and sweet cherry (*Prunus avium*) [7]. Various studies have been carried out to elucidate the mechanisms underlying fruit cracking.

Previous studies have shown that excess water absorbed by roots or directly by the fruit surface are responsible for fruit cracking [8]. In some studies, treatment of fruit surface with liquid water has been reported to increase fruit cracking [5]. Cracking of the fruit caused by rainfall before harvest is a serious problem in some fruit species, including cherries and grapes [9]. Several researchers have reported that prevention of rainwater uptake through the cuticle can reduce cherry cracking [7]. The relationship between fruit cracking and changes in levels of irrigation has been studied in nectarine and litchi. High water supply triggers high incidence of fruit cracking in nectarine [6] and litchi [10].

Researchers have found a positive correlation between fruit osmotic potential and fruit cracking [11], which has led to the proposition that absorption of water by fruits is attributable to osmotic potential and that excess water inside fruits causes them to crack. Lu and Lin [3] found that water enters the fruit in response to decreased tissue osmotic potential.

Gibberellin (GA) hormones act throughout the life cycle of plants, influencing seed germination, stem elongation, flower induction, anther development, and seed and pericarp growth. GA is widely used in various horticultural crops for improving fruit set and controlling fruit cracking. Hoda and Khalil [2] found that application of GA_3_ (80 ppm) reduced cracking in pomegranate (*Punica granatum* L.). Cline and Trought [12] reported that application of GA_3_ reduced cracking in cherry. In litchi, Munish *et al.* [13] demonstrated that spraying of GA_3_ (25 and 50 ppm) reduced fruit cracking.

Abscissic acid (ABA) is a phytohormone that regulates various processes of plant development, such as seed dormancy and senescence. ABA also functions in adaptation to abiotic stresses. Li *et al.* [11] found that application of ABA increased fruit cracking in litchi. Yilmaz and Ozguven [14] found that the ABA content of the peel is higher in cracked fruits than in the peel of healthy (non-cracked) fruits. Sharma and Dhillon [15] observed that ABA content in pericarp is higher in cracking fruits than in the healthy fruits.

Calcium is one of the most important components that provide strength to cell walls. Low calcium concentration in pericarp cells is correlated with fruit cracking in tomato (*Solanum lycopersicum*) [16] and litchi (*Litchi chinensis* Sonn.) [17]. Hoda and Khalil [2] found that application of calcium chloride (3%) reduced cracking in pomegranate (*Punica granatum* L.). Haq [10] found that application of CaCl_2_ increased calcium content and decreased fruit cracking in litchi.

Previous studies have indicated that pectin esterase (PE) and polygalacturonase (PG) are the key enzymes involved in the process of plant pectin degradation, and cellulose (EG) is one of the key enzymes involved in cellulose degradation. Expansins (EXP) are cell wall proteins that facilitate extension of cell walls, and are considered to be primary regulators of plant cell enlargement. β-galactosidases (β-Gal) reduce the levels of cell wall galactosyl residues in ripening tomato fruits. Xyloglucan endotransglycosylase (XET) catalyses the transglycosylation of xyloglucan, the major hemicelluloses polymer, which is thought to mediate cross-linking of cellulose microfibrils in cell walls and is proposed to be involved in the control of cell wall relaxation [18].

Investigations of mechanisms underlying fruit cracking in sweet cherry has revealed that a combination of physiological, biochemical, genetics and molecular factors is involved [7]. Research on litchi fruit cracking has focused mostly on biochemical analysis of nutrients [10], water [10,11,17] and hormones [11,13,15], structure and mechanical properties of the pericarp [10], enzymes involved [19], and girdling [11]. However, very few studies have focused on molecular factors. Only *LcEXP1* [20], *LcEXP2* [20] and *LcXET1* [18] have been reported to be associated with fruit cracking to date. The present study aimed to identify other important genes associated with litchi fruit cracking and to explain the molecular relationship between litchi fruit cracking and water transport, GA metabolism, ABA metabolism, Ca transport and cell wall metabolism. The transcriptome of cracking pericarp of litchi was first assembled *de novo* and annotated using RNA-Seq. Various computational tools were then used to identify genes related to pericarp and fruit cracking in litchi.

## 2. Results

### 2.1. cDNA Sequence Generation and de Novo Assembly

Pooled cDNA samples representing two groups of litchi pericarp were prepared and sequenced using the Illumina Genome Analyser HiSeq 2000. The RNA-Seq data were subjected to bioinformatic analysis (Figure S1a–c). After quality checking and data cleaning, a total of approximately 55 million (~25 and ~29 million for samples 1 and 2, respectively) 100 bp paired-end reads were obtained (Table 1). Assembly of reads produced 42,790 and 42,447 contigs, with average lengths of 966 and 967 bp for samples 1 and 2, respectively. After paired-end joining and gap-filling, 38,917 unigenes were produced for sample 1, with an average length of 949 bp, whereas 38,695 unigenes were produced for sample 2, with an average length of 950 bp (Table 1). The size distributions of contigs and unigenes are shown in Figure S2.

**Table 1:** Summary of the litchi pericarp transcriptome.

Category	Sample 1	Sample 2
Number of reads	25,867,380	29,444,512
Average read length (bp)	100	100
Total length of reads (bp)	2,586,738,000	2,944,451,200
Number of contigs	42,790	42,447
Average length of contigs (bp)	966	967
Number of unigenes	38,917	38,695
Average length of unigenes (bp)	949	950
Total length of unigenes	36,945,798	36,761,221

### 2.2. Functional Annotation of the Transcriptome in Litchi Pericarp

For annotation, the assembled unigenes were compared against several protein databases (Nr, SwissProt, KEGG and COG) using BLASTX (<E-value 10^−5^). The results revealed that 32,640 of the 46,641 unigenes (69.98%) were successfully annotated in these databases, and 32,479 of the 46,641 unigenes (69.64%) had significant matches in the NR database (Table 2). The overall functional annotation is depicted in Table S1.

**Table 2:** Hit percentages against important public databases.

Public Protein Database	Number of Unigene Hits	Percentage (%) *
NCBI NR	32,479	69.64
Swiss-Prot	25,010	53.62
COG	12,981	27.83
KEGG	9504	20.38

* Proportion of the 46,641 assembled unigenes.

**Figure 1:**
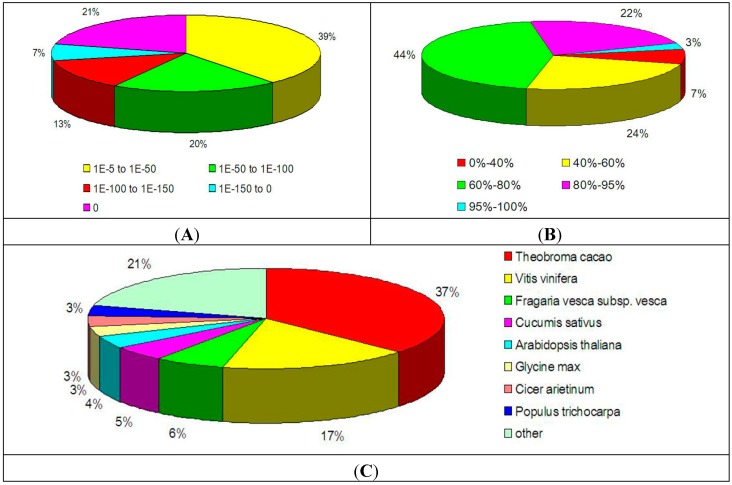
Characteristics of homology search of litchi pericarp unigenes against the NR database. (**A**) E-value distribution of the top BLAST hits for each unique sequence; (**B**) Similarity distribution of the top BLAST hits for each unique sequence; (**C**) Species distribution of the top BLAST hits for all homologous sequences.

The E-value, similarity and species distributions are described in Figure 1 to further analyze the BLAST results. Statistical analysis of the top hits in the NR database indicated that 61% of the matched sequences showed strong homology (<1.0 E^−50^), whereas 39% of the mapped sequences showed moderate homology (between 1.0 E^−5^ and 1.0 E^−50^) (Figure 1A). The similarity distribution pattern indicated that 25.0% of the sequences had similarity higher than 80%, whereas 75.0% showed similarity between 0 and 80% (Figure 1B). The species distribution pattern showed that 37.0% of the unique sequences had top matches to sequences from *Theobroma cacao*, with additional hits to *Vitis vinifera* (17.0%), *Fragaria vesca subsp. vesca* (7.0%), *Cucumis sativus* (5.0%) and *Arabidopsis thaliana* (4.0%) (Figure 1C).

### 2.3. GO Annotation

**Figure 2:**
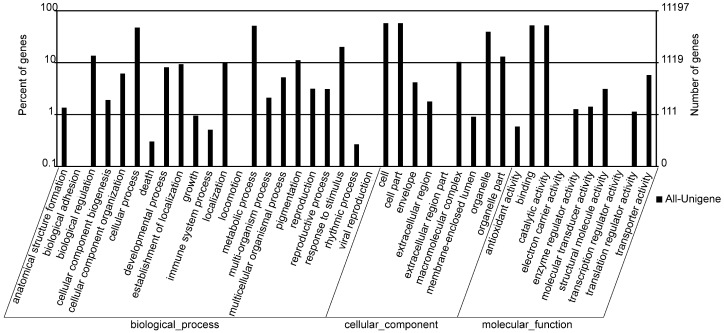
Gene Ontology (GO) classification for all unigenes. All unigenes were aggregated into three main categories: biological process, cellular component and molecular function. Percentages are based on the proportion and number of genes in each set.

A total of 56,019 unigenes were categorized into 41 functional groups using Gene Ontology (GO) analysis. The unigenes were distributed to the following three main categories: biological process (22,007), cellular components (20,791) and molecular function (13,221) (Figure 2 and Table S2). Within the biological process category, the metabolic processes (26.03%) and the cellular process (24.13%) were the top two most represented GO terms. The cell (33.18%), cell part (33.18%) and organelle (21.25%) were enriched in the cellular components category. Under the molecular function category, genes encoding binding proteins (44.47%) and proteins related to catalytic activity (44.18%) were the most highly represented GO terms.

### 2.4. COG Annotation

**Figure 3:**
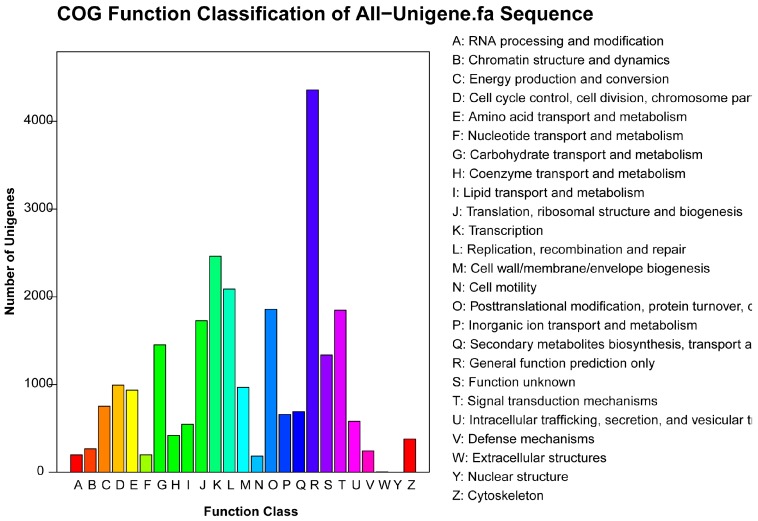
Clusters of Orthologous Groups (COG) function classification of all-unigene sequence. A total of 25,166 unigenes were classified into 25 COG categories.

A total of 25,166 unigenes were classified into 25 COG categories (Figure 3 and Table S3) using Clusters of Orthologous Groups (COG) analysis. The general function prediction (4358; 17.32%) represented the largest group, followed by transcription (2464; 9.79%), replication, recombination and repair (2089; 8.30%), posttranslational modification, protein turnover, chaperones (1858; 7.38%), signal transduction mechanisms (1847; 7.34%), translation, ribosomal structure and biogenesis (1729; 6.87%), carbohydrate transport and metabolism (1453; 5.77%) and so on. The extracellular and nuclear structures comprised the smallest groups (5 and 1 unigenes, respectively).

### 2.5. KEGG Pathway Mapping

**Figure 4:**
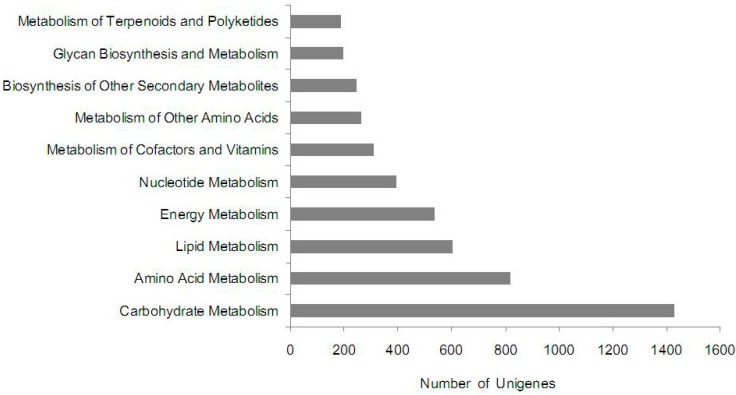
Pathway assignment based on the Kyoto Encyclopedia of Genes and Genomes (KEGG).

Referencing the 9504 unigenes predicted a total of 123 pathways using the Kyoto Encyclopedia of Genes and Genomes (KEGG) analysis, representing compound biosynthesis, degradation, utilization and metabolism. Of these, about 4992 unigenes were significantly enriched in 10 metabolic pathways (Figure 4 and Table S4). The top represented pathway was carbohydrate metabolism (1430; 28.65%), followed by amino acid metabolism (817; 16.37%), lipid metabolism (604; 12.10%), energy metabolism (537; 10.76%), and nucleotide metabolism (397; 7.95%).

### 2.6. Differentially Expressed Unigenes between Non-Cracking Fruit and Cracking Fruit Libraries

**Figure 5:**
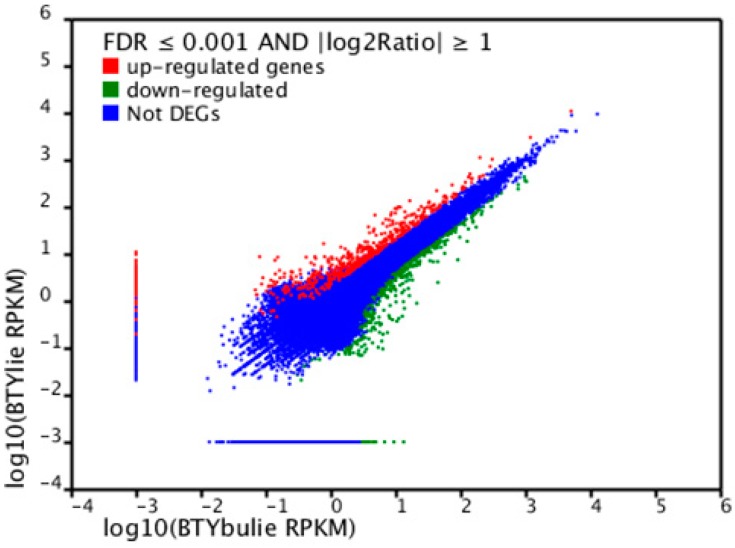
Comparison of transcript abundance levels between samples 1 and 2. Differentially expressed genes are shown in red and green; blue indicates genes that were not differentially expressed between the two samples.

The present study compared the two samples by differential expression analysis. A total of 1998 unigenes showed significant differential expression (false discovery rate [FDR] ≤ 0.001, |log2 ratio| ≥ 1, Table S5) between the two samples. Among these unigenes, 632 genes were upregulated in cracking fruits compared with non-cracking fruits. Meanwhile, the expression of 1366 genes was downregulated in cracking fruits compared with non-cracking fruits (Figure 5 and Table S5). The differentially expressed unigenes were also subjected to GO analysis and categorized into 37 GO functional groups (Figure S3a). In addition, these unigenes were also subjected to KEGG pathway enrichment analysis, and 516 unigenes were found to be significantly enriched in 19 pathways (Figure S3b and Table S6).

### 2.7. Identification of Candidate Genes for Fruit Cracking in Litchi chinensis Sonn.

**Figure 6:**
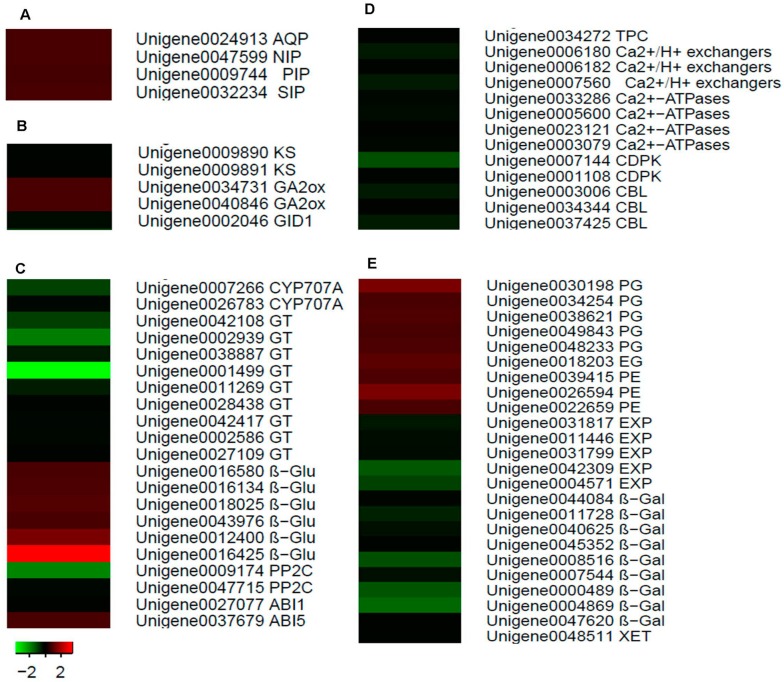
Heat map diagram of candidate genes for fruit cracking in *Litchi chinensis* Sonn. The DEGs annotated in pathways related to water transport (**A**); gibberellin (GA) metabolism (**B**); abscissic acid (ABA) metabolism (**C**); Ca transport (**D**); cell wall metabolism (**E**) are shown.

A total of 67 genes related to fruit cracking were identified. These genes are involved in water transport (4 genes), GA metabolism (5 genes), ABA metabolism (21 genes), Ca transport (13 genes), and cell wall metabolism (24 genes). The pathways were then analyzed using a heat map (Figure 6 and Table S7).

The results showed that four genes, namely *LcAQP*, *LcPIP*, *LcNIP* and *LcSIP*, were upregulated two-fold in cracking fruits compared to normal fruits. All four of these genes are related to water transport (Figure 6A and Table S7).

Two *LcKS* genes and one *LcGID1* gene, all of which are involved in GA metabolism, were expressed more than two-fold in normal fruits compared to cracking fruits. Meanwhile, two *LcGA2ox* genes were upregulated more than two-fold in cracking fruits compared to normal fruits (Figure 6B and Table S7).

Of the genes involved in ABA metabolism, two *LcCYP707A* genes were found to be upregulated two-fold and five-fold, respectively, in normal fruits compared to cracking fruits. Nine *LcGT* genes were upregulated between two-fold and seventeen-fold in normal fruits compared to cracking fruits. Six *Lcβ-Glu* genes were upregulated between two-fold and twelve-fold in cracking fruits compared to normal fruits. Two *LcPP2C* and one *LcABI1* genes were upregulated two-fold to nine-fold in normal fruits compared to cracking fruits. One *LcABI5* gene was upregulated more than two-fold in cracking fruits compared to normal fruits (Figure 6C and Table S7).

Among genes involved in Ca transport, one *LcTPC* gene was upregulated two-fold in normal fruits compared to cracking fruits. Three *Ca^2+^/H^+^ exchanger* genes were upregulated two-fold to three-fold in normal fruits compared to cracking fruits. Four *Ca^2+^-ATPase* genes were upregulated more than two-fold in normal fruits. Two *LcCDPK* genes were upregulated two-fold to six-fold in normal fruits compared to cracking fruits. Three *LcCBL* genes were upregulated two-fold to three-fold higher in normal fruits relative to cracking fruits (Figure 6D and Table S7).

Of the genes involved in cell wall metabolism, five *LcPG*, one *LcEG* and three *LcPE* genes were upregulated between two-fold and four-fold in cracking fruits compared to normal fruits. Meanwhile, five *LcEXP*, nine *Lcβ-Gal* and one *LcXET* genes were upregulated between two-fold and seven-fold in normal fruits compared to cracking fruits (Figure 6E and Table S7).

## 3. Discussion

### 3.1. First Litchi Cracking Pericarp Reference Transcriptome Generated by RNA-Seq

Many researchers have indicated that SOAP *de novo* is a useful tool for annotating transcriptomes of plant species [21]. In the current study, a total of 46,317,478 bp were sequenced and assembled into a reference transcriptome of litchi pericarp. This is the first report of a cracking pericarp library for the Sapindaceae family. A total of 46,641 unigenes (≥200 bp) were identified. The mean unigene size was 993 bp, which was longer than that reported in other studies using a similar approach, such as 601 bp by Li *et al.* [21]. More than 69% of unigenes identified in the present study matched the NR database, which was comparable with the results of other studies using the same technology (e.g., 59% by Li *et al.* [21]). These results indicate that the litchi pericarp transcriptome presented here is comprehensive, accurate, and useful for future genetic research of litchi pericarp and pericarp of other Sapindaceae family plant species.

### 3.2. Identification of Candidate Genes for Fruit Cracking in Water Transport

Previous studies have shown that excess water is the cause of fruit cracking [8]. Aquaporins (AQP) are known to mediate water transport in cells. Flexas *et al.* [22] found that plants in which *NtAQP1* is overexpressed exhibit 20% increase in photosynthetic rate compared to wild type controls, which may be due to higher rate of water transfer in the former. Furthermore, they found that *NtAQP1* antisense plants showed 13% decrease in photosynthetic rate, which may be due to reduced water transport. In the present study, one *LcAQP* gene was observed to be upregulated in cracking fruits. Aharon *et al.* [23] constitutively overexpressed the Arabidopsis plasma membrane aquaporin PIP1b in transgenic tobacco plants. They found that this resulted in transfer of more water, increasing the transpiration and water consumption rates. In the present study, one *LcPIP* gene was upregulated in cracking fruits. Murai-Hatano *et al.* [24] reported that root hydraulic conductivity decreased with decreasing root temperature. Matsumoto *et al.* [25] showed that expression of *OsNIP2;1*, *OsSIP1;1* and *OsSIP2;1* decreased with decreasing temperature. Notably, one *LcNIP* gene and one *LcSIP* gene were found to be upregulated in cracking fruits in the present study.

Totally, 36 differential expression genes were identified involved in water transport in the present study (Table S8). Among them 15 genes were up-regulated, but 21 genes were down-regulated (7 genes down-regulated more than two-fold) in cracking fruit. Not all of these differential expression genes involved in water transport were up-regulated in cracking fruit. The roles of these down-regulated genes involved in water transport and water content need future research. Meanwhile, we selected 10 genes involved in water transport to qRT-PCR in three biological replicates (Figure S4B). The qRT-PCR results were strictly consistent with transcriptome analysis.

Based on the results described above, upregulated expression of *LcAQP*, *LcPIP*, *LcNIP* and *LcSIP* genes in pericarp is hypothesized to facilitate entrance of excess water into aril cells, which can cause cells to swell, thereby producing more turgor pressure against the skin and eventually resulting in skin rupture. Thus, *LcAQP*, *LcPIP*, *LcNIPs* and *LcSIPs* genes may be the key genes controlling litchi fruit cracking via regulation of water transport.

### 3.3. Identification of GA Metabolism-Related Candidate Genes for Fruit Cracking

Previous studies have shown that application of gibberellins can reduce fruit cracking [12,13] because of increase in GA levels in fruits. The ent-kaurene synthase (*KS*) gene encodes the enzyme responsible for catalysis of the second step in the GA biosynthesis pathway. In the present study, two *LcKS* genes were found to be downregulated in cracking fruits. Gibberellin 2-oxidase (*GA2ox*) has a key role in the GA catabolic pathway through 2β-hydroxylation. Previous studies have shown that GA 2-oxidase negatively regulates the GA levels [26]. Schomburg *et al.* [26] reported that increased expression of either *AtGA2ox7* or *AtGA2ox8* can reduce GA levels. In the present study, expression of two *LcGA2ox* genes were monitored and found to be upregulated in cracking fruits. It has been shown that expression of GIBBERELLIN INSENSITIVE DWARF1 (*GID1*), the receptor of GA, is positively correlated with GA levels. Overexpression of *GID1* rescued the dwarf phenotype of sly1 and gid2 mutants by altering GA levels [27]. Notably, one *LcGID1* gene was found to be downregulated in cracking fruits in the present study.

Based on the abovementioned results, we propose that downregulated expression of *LcKS* and *LcGID1* genes in the pericarp and upregulated expression of *LcGA2ox* genes, which may lead to lower GA level in the pericarp, cause fruit cracking. Therefore, *LcKS*, *LcGID1* and *LcGA2ox* genes are likely the key GA metabolism-related genes controlling litchi fruit cracking.

### 3.4. Identification of Candidate ABA Metabolism-Related Genes for Fruit Cracking

Previous studies have shown that higher ABA levels in pericarp is correlated with fruit cracking [14,15]. ABA 8'-hydroxylation may have a predominant role in ABA catabolism, and *CYP707As* encodes ABA 8'-hydroxylases. Kushiro *et al.* [28] found that expression of *CYP707A2* increased with decreasing levels of ABA during seed imbibition. Furthermore, Kushiro *et al.* [28] found that the ABA content was six-fold higher in cyp707a2 mutants than wild type. In the present study, two *LcCYP707A* genes were found to be downregulated in cracking fruits. ABA is conjugated with glucose by ABA glycosyltransferase (GT), forming the physiologically inactive ABA glucosyl ester [29]. Nine *LcGT* genes were also found to be downregulated in cracking fruits. *AtBG1*, a β-glucosidase (β-Glu), hydrolyses glucose-conjugated, biologically inactive ABA to produce active ABA. *AtBG1* contributes to accumulation of ABA, and loss of *AtBG1* causes lower ABA levels [29]. In the present study, six *Lcβ-Glu* genes were upregulated in cracking fruits. Protein phosphatase 2C (PP2C) acts as a negative regulator of ABA signaling. Furthermore, *ABI1* belongs to the *PP2C* family of genes. Previous studies have revealed that *ABI1* negatively regulates the ABA response in Arabidopsis [30]. In the present study, two *LcPP2C* and one *LcABI1* genes were found to be downregulated in cracking fruits. *ABI5* belongs to the ABF gene family. Finkelstein and Lynch [31] found that *ABI5* is a positive regulator of ABA signaling. Considering these facts, it is notable that one *LcABI5* gene was found to be upregulated in cracking fruits in the present study.

In summary, upregulated expression of *Lcβ-Glu* and *LcABI5*, and downregulated expression of *LcCYP707A*, *LcGT*, *LcPP2C* and *LcABI1* genes in pericarp, which can then lead to higher ABA level in pericarpand eventually to fruit cracking in litchi. Thus, *LcCYP707A*, *LcGTs*, *Lcβ-Glu*, *LcPP2C*, *LcABI1* and *LcABI5* may be the key ABA metabolism-related genes that control litchi fruit cracking.

### 3.5. Identification of Candidate Ca Transport-Related Genes for Fruit Cracking

Previous studies have shown that low Ca content in the pericarp is related to fruit cracking [16], and spraying calcium can reduce fruit cracking [2,10] by increasing Ca content in the pericarp. To regulate Ca^2+^ concentrations in cell compartments, plants utilize Ca^2+^ channels, Ca^2+^/H^+^ exchanger, Ca^2+^-ATPase and calcium-binding proteins. TPC acts as a putative voltage-gated Ca^2+^ channel that can regulate Ca^2+^ transport [32]. Furuichi *et al.* [32] suggested that a cytosolic-free Ca^2+^ increase in aequorin-expressing Arabidopsis leaves was enhanced by overexpression of *AtTPC1* and suppressed by expression of its antisense. In the present study, one *LcTPC* gene was downregulated in cracking fruits. CAX proteins have been characterized as Ca^2+^/H^+^ exchanger [33]. Kamiya *et al.* [33] found that rice treated with 100 mM CaCl_2_ can increase *OsCAX1a* mRNA levels. *OsCAX1a* mRNA levels in roots were approximately twice that of control levels. In the present study, three litchi *Ca^2+^/H^+^ exchanger* genes were found to be downregulated in cracking fruits. Previous studies have shown that cytoplasmic Ca^2+^ levels in plant cells increase rapidly in response to multiple stress stimuli, including cold temperature, high salt level and drought. Perez-Prat *et al.* [34] revealed that several putative P-type Ca^2+^-ATPase are induced by sodium (Na^+^) stress. In the present study, four litchi *Ca^2+^-ATPase* genes were found to be downregulated in cracking fruits. Calcium-binding proteins include calmodulin proteins (CaM), calcineurin B-like protein (CBL) and Ca^2+^-dependent protein kinases (CDPK). Pandey *et al.* [35] found that expression of the *CBL9* gene in seedlings is highly inducible by salt (300 mM NaCl), drought (dehydration) and cold. In addition, the *CBL1* gene transcript was induced by drought, cold and wounding. Saijo *et al.* [36] found that *OsCDPK7* mRNA levels increased under cold- and salt-stress conditions in both shoots and roots. In the present study, two *LcCDPK* and three *LcCBL* genes were found to be downregulated in cracking fruits.

Based on the above results, downregulated expression of *LcTPC*, *Ca^2+^/H^+^ exchanger*, *Ca^2+^-ATPase*, *LcCDPK* and *LcCBL* genes is likely to lead to lower Ca content in pericarp, thereby degrading the structure of the skin and leading to litchi fruit cracking. Therefore, *LcTPC*, *Ca^2+^/H^+^ exchanger*, *Ca^2+^-ATPase*, *LcCDPK* and *LcCBL* genes are putative key genes controlling litchi fruit cracking.

### 3.6. Identification of Candidate Cell Wall Metabolism Genes for Fruit Cracking

Lu and Lin [4] reported that fruit cracking was accompanied by increased PG enzyme activity in wax apple. Brummell *et al.* [37] suggested that antisense inhibition of PE and PG activity in tomato reduced fruit cracking. Li *et al.* [19] found that the activity of PG and EG is higher in Nuomici (cracking-susceptible) pericarp than in Huaizhi (cracking-resistant) pericarp. Schuch *et al.* [38] decreased *PG* and *PE* levels by transgenic antisense technology and found that doing so significantly reduced cracking in tomato. Amita *et al.* [39] found that accumulation and activities of *EG* transcripts increased upon ethylene treatment. In the present study, five *LcPG*, one *LcEG* and three *LcPE* genes were upregulated in cracking fruits.

Balbontín *et al.* [40] studied sweet cherry cultivars with different degrees of susceptibility to cracking at different stages of fruit development, and found that expression of the *expansin* gene is higher in the more resistant cultivar than in the susceptible cultivar. Wang *et al.* [20] isolated two *expansin* genes, namely *LcEXP1* and *LcEXP2*, in litchi and compared their expression levels in two difference cultivars: the cracking-resistant cultivar Huaizhi and the cracking-susceptible cultivar Nuomici. Results of their study indicated that *LcExp1* mRNA levels are higher in Huaizhi pericarp than in Nuomici pericarp. *LcEXP2* mRNA levels can only be detected in pericarp of Huaizhi, but not in Nuomici pericarp. In the present study, we found that expression of five *LcEXP* genes were down-regulated in cracking fruits.

Knoche *et al.* [41] studied sweet cherry cultivars with different degrees of susceptibility to cracking and discovered that expression of the β-galactosidase (*β-Gal*) gene was higher in the more resistant cultivar (“Kordia”) than in the susceptible cultivar (“Bing”). Moctezuma *et al.* [42] found that antisense suppression of *galactosidase* gene expression in tomato increases fruit cracking. Considering these findings, it is notable that nine *Lcβ-Gal* genes were found to be downregulated in cracking fruits in the present study.

Lu *et al.* [18] reported that enhanced *LcXET1* mRNA accumulation in pericarp could reduce litchi fruit cracking. Consistent with this result, we found one *LcXET* gene to be downregulated in cracking fruits in the present study.

Based on the above results, we propose that upregulation of *LcPG*, *LcEG* and *LcPE* genes in pericarp increase the activity of PG, EG and PE, thereby degrading the structure of the skin. Furthermore, downregulated expression of *LcEXP*, *Lcβ-Gal* and *LcXET* genes in pericarp can reduce elasticity of the skin, which eventually leads to litchi fruit cracking. Thus, *LcPG*, *LcEG*, *LcPE*, *LcEXP*, *Lcβ-Gal* and *LcXET* genes may be the key cell wall metabolism genes that are involved in litchi fruit cracking.

## 4. Experimental Sections

### 4.1. Plant Materials

Litchi trees were selected in an orchard in Leizhou, Zhanjiang city, China. Six 10-year-old “Baitangying” trees (in 2013, with the same genotypes) with uniform vigor and moderate initial crop load were chosen for sample collection. Cracking and non-cracking fruits were randomly taken from two trees as a biological replicate. Totally, there were three biological replicates. The RNA was extracted from pericarp of non-cracking and cracking fruit in each replicate, then pooled with equivalent quantities RNA from the three replicate to form sample 1 (BTYbulie) RNA and sample 2 (BTYlie) RNA. Pericarp was collected immediately after the samples were transported back to the laboratory on ice. After separation, all tissues were quickly frozen in liquid nitrogen and stored at −80 °C for future analysis.

### 4.2. RNA Isolation and Library Construction for Transcriptome Analysis

Total RNA was isolated from frozen pericarp sample according to the methods of Wu *et al.* [43]. DNase I (TaKaRa, Otsu, Japan) was added to remove potentially contaminating DNA, and RNAse-free columns (Huayueyang, Beijing, China) were used for purifying total RNA. The 2100 Bioanalyzer (Agilent Technologies, Santa Clara, CA, USA) was used to confirm the integrity of RNA. The workflow for library construction for transcriptome analysis is described in Figure S1a. In summary, after RNA was collected, poly(A)-containing mRNA was purified using oligo(dT) magnetic beads. Fragmentation buffer was used to chop the mRNA into short fragments, which were then used as templates for random hexamer-primed synthesis of the first strand cDNA. Afterwards, DNA polymerase I and RNAse H were used for second-strand cDNA synthesis. The synthesized cDNA fragments were subjected to end repair, addition of a single “A” base, ligation with adapters and purification and amplification through PCR to construct the sequencing library.

### 4.3. Transcriptome Sequencing, de Novo Assembly and Functional Annotation

The overall workflows of the transcriptome assembly and bioinformatic analysis are shown in Figure 1b,c, respectively. Solexa HiSeq™ 2000 was used to sequence the library (BGI, Shenzhen, China). The obtained raw reads were pre-processed by removing the adaptor sequences, resulting in clean reads. The short reads were first assembled into contigs using the SOAPdenovo assembly program, and unigenes were obtained. The assembled unigenes were aligned to a series of protein databases by BLASTx alignment (E-value < 0.00001). These databases include the NCBI NR protein database, the Swiss-Prot protein database, the KEGG pathway database and the COG database. Sequence direction of unigenes was assigned according to the best alignments from the four databases. When different databases showed different results, the following order of priority was employed: NR, Swiss-Prot, KEGG and COG. When unigenes failed to be annotated through any one of the abovementioned databases, the ESTScan program was employed to predict the “CDS” (coding regions) and orientations. Blast2GO software was used to annotate unigene GO function. After GO functional classification, the WEGO software was used to view the distribution of gene functions in Litchi pericarp at the macro level.

### 4.4. Identification of Differentially Expressed Unigenes

The SOAP2 software was used to analyse unigene transcript abundance levels between the two samples (non-cracking fruit and cracking fruit), and the RPKM (Reads Per Kb per Million reads) values were calculated as proposed by Mortazavi *et al.* Unigene transcript abundance differences between the two samples of litchi pericarp were obtained from RPKM values based on a method modified from Audic’s proposal. Fold changes for each unigene between sample pairs (sample 2 *vs.* sample 1) were calculated as the ratio of the respective RPKM values. When the value of sample 2 RPKM or sample 1 RPKM was 0, the value 0.001 was used instead of 0 to determine the fold change. The significance of unigene expression differences was calculated using the FDR (False Discovery Rate) control method to justify the p-value, and only unigenes with an absolute value of log2 ratio ≥ 1 (fold-change ≥ 2) and a FDR ≤ 0.001 were used as thresholds to determine the statistical significance of unigene expression differences. The formula to determine the significant p-value between two samples was defined as follows.

(1) $$p(i|x)={\left(\frac{N_{2}}{N_{1}}\right)}^{i}\frac{(x+i)!}{x!i!{\left(1+\frac{N_{2}}{N_{1}}\right)}^{\left(x+i+1\right)}}$$

In the formula, N1 and N2 represent the total number of clean reads mapped to All-unigenes in each sample, and x and *i* represent respectively the number of clean reads mapped to a common unigene in sample 1 and sample 2.

## 5. Conclusions

**Figure 7:**
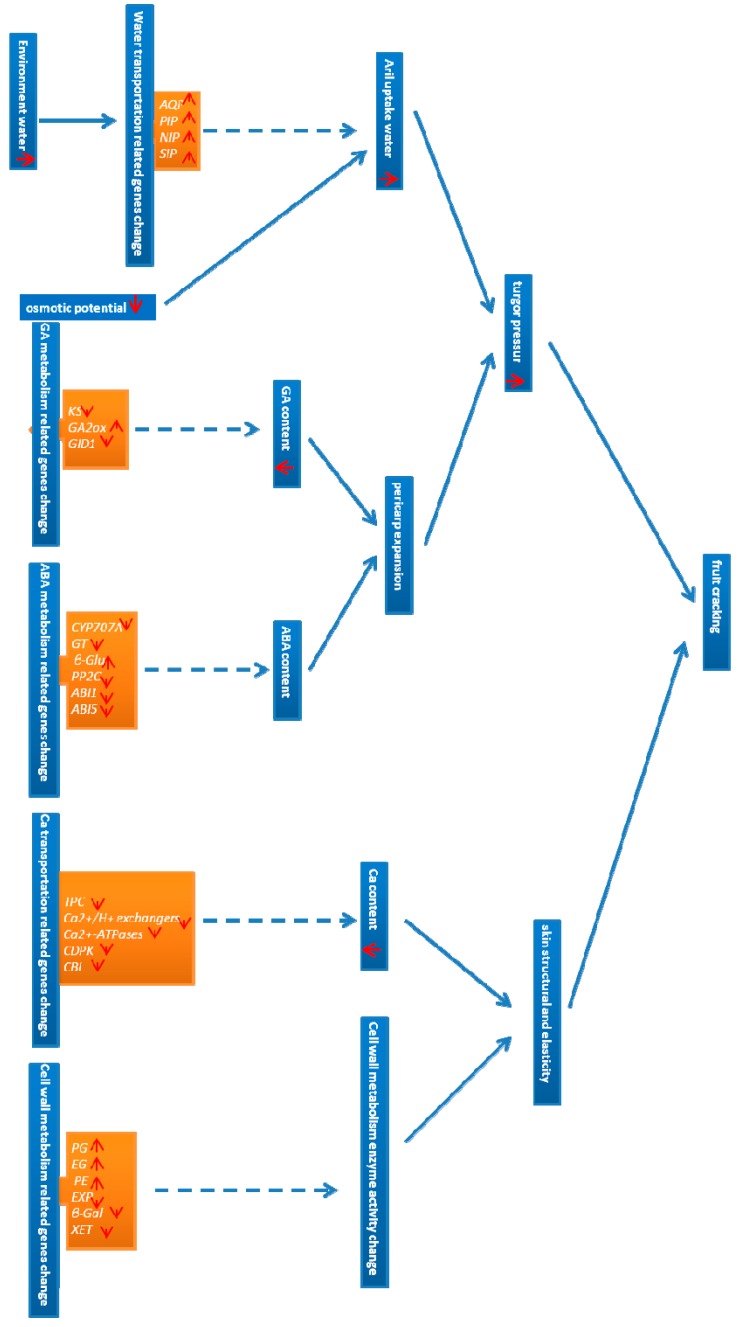
Hypothesized mechanisms of fruit cracking in litchi.

In the present study, the mechanisms underlying litchi fruit cracking were hypothesized to be turgor pressure, structure and elasticity of skin. Fruit cracking does not occur when these three mechanisms are balanced. However, when aril produces more turgor pressure against the skin or reduces the structure and elasticity of the skin, fruit cracking occurs (Figure 7).

Increased turgor pressure may be caused by two factors. One is the excessive entrance of water into aril cells, which can cause them to swell. As a result, aril produces more turgor pressure against the skin, thereby resulting in skin rupture. Therefore, when subjected to rainy conditions, fruits take up more water, thereby resulting in fruit cracking. In addition, lower osmotic potential also increases the entrance of water into the fruit, which triggers fruit cracking. However, some agronomic measures can be undertaken to reduce water on the surface of the fruit to decrease fruit cracking. In the present study, four genes (*LcAQP*, *LcPIP*, *LcNIP* and *LcSIP*) involved in water transport were identified as candidate genes that mediate fruit cracking of litchi. The second factor is lower GA levels and higher ABA levels in pericarp, which leads to slower expansion of the pericarp, so that aril cells produce more turgor pressure against the skin, thereby leading to fruit cracking. These findings are supported by many researchers who have found that spraying GA can reduce fruit cracking but spraying ABA can increase fruit cracking. Moreover, many researchers have observed that GA contents are higher in cracked fruits than in healthy fruits. In the present study, five genes (*LcKS*, 2; *LcGA2ox*, 2; *LcGID1*, 1) involved in GA metabolism and 21 genes (*LcCYP707A*, 2; *LcGT*, 9; *Lcβ-Glu*, 6; *LcPP2C*, 2; *LcABI1*, 1; *LcABI5*, 1) involved ABA metabolism were identified as candidate genes for fruit cracking of litchi.

The structure and elasticity of litchi fruit skin can be reduced by two factors. One is decreased calcium content, which degrades the skin structure and thus leads to fruit cracking. Consistent with this, spraying calcium has been found to reduce fruit cracking. In the present study, 13 genes (*LcTPC*, 1; *Ca^2+^/H^+^ exchanger*, 3; *Ca^2+^-ATPase*, 4; *LcCDPK*, 2; *LcCBL*, 3) involved in Ca^2+^ transport were identified as candidate genes for fruit cracking of litchi. The other factor is that gene expression changes lead to changes in enzyme activity related to cell wall metabolism. As a result, the structure and elasticity of the skin is reduced, resulting in fruit cracking. Consistent with this idea, many researchers have observed that fruit cracking is positively correlated with increased expression of *PG* gene, and that increased enzyme activity of PG, EG, and PE are related to deteriorated skin structure. Furthermore, expression levels of *EXP*, *β-Gal* and *XET* were found to be decreased in cracking fruits, which likely contributes to reduced skin elasticity. In the present study, 24 genes (*LcPG*, 5; *LcEG*, 1; *LcPE*, 3; *LcEXP*, 5; *Lcβ-Gal*, 9; *LcXET*, 1) involved in cell wall metabolism were identified as candidate genes for fruit cracking in litchi.

Based on the results of this study, a predictable molecular model could be established to explain the mechanism underlying fruit cracking in litchi. The understanding of how water, gibberellins, abscisic acid, calcium and the cell wall biosynthesis affect litchi fruit cracking will facilitate the development of an integrated agricultural practice to reduce fruit cracking rate. And furthermore, fruit cracking also occur in tomato, pomegranate, apple, nectarine and so on. Although the fruit skin (pericarp) structure of litchi is different with other fruit species, the results of this study may provide a reference for other researchers.

## Supplementary Materials

Supplementary materials can be found at http://www.mdpi.com/1422-0067/15/10/17667/s1.

## Supplementary Files

Supplementary File 1
